# Lessons on the Sigma-1 Receptor in TNBS-Induced Rat Colitis: Modulation of the UCHL-1, IL-6 Pathway

**DOI:** 10.3390/ijms21114046

**Published:** 2020-06-05

**Authors:** Nikoletta Almási, Szilvia Török, Szabolcs Dvorácskó, Csaba Tömböly, Ákos Csonka, Zoltán Baráth, Zsolt Murlasits, Zsuzsanna Valkusz, Anikó Pósa, Csaba Varga, Krisztina Kupai

**Affiliations:** 1 Department of Physiology, Anatomy and Neuroscience, University of Szeged, H-6726 Szeged, Hungary; almasi@expbio.bio.u-szeged.hu (N.A.); tszilvia@bio.u-szeged.hu (S.T.); paniko@bio.u-szeged.hu (A.P.); vacs@bio.u-szeged.hu (C.V.); 2 Laboratory of Chemical Biology, Institute of Biochemistry, Biological Research Centre, H-6726 Szeged, Hungary; dvoracsko.szabolcs@brc.hu (S.D.); tomboly.csaba@brc.hu (C.T.); 3 Department of Medical Chemistry, University of Szeged, H-6725 Szeged, Hungary; 4 Department of Traumatology, University of Szeged, H-6725 Szeged, Hungary; csonka.akos81@gmail.com; 5 Department of Orthodontics and Pediatric Dentistry, Faculty of Dentistry, University of Szeged, H-6720 Szeged, Hungary; barzol34@gmail.com; 6 Laboratory Animals Research Center, Qatar University, Doha 2713, Qatar; zmrlsits@me.com; 7 1st Department of Medicine, Medical Faculty, Albert Szent-Györgyi Clinical Center, University of Szeged, H-6720 Szeged, Hungary; valkusz.zsuzsanna@med.u-szeged.hu

**Keywords:** sigma receptor, fluvoxamine, inflammation, UCHL-1

## Abstract

Inflammatory Bowel Disease (IBD) is an autoimmune ailment of the gastrointestinal (GI) tract, which is characterized by enhanced activation of proinflammatory cytokines. It is suggested that the sigma-1 receptor (σ1R) confers anti-inflammatory effects. As the exact pathogenesis of IBD is still unknown and treatment options are limited, we aimed to investigate the effects of σ1R in 2,4,6-trinitrobenzenesulfonic acid (TNBS)-induced experimental colitis. To this end, male Wistar–Harlan rats were used to model colitic inflammation through the administration of TNBS. To investigate the effects of σ1R, Fluvoxamine (FLV, σ1R agonist) and BD1063 (σ1R antagonist) were applied via intracolonic administration to the animals once a day for three days. Our radioligand binding studies indicated the existence of σ1Rs as [^3^H](+)-pentazocine binding sites, and FLV treatment increased the reduced σ1R maximum binding capacity in TNBS-induced colitis. Furthermore, FLV significantly attenuated the colonic damage, the effect of which was abolished by the administration of BD1063. Additionally, FLV potentially increased the expression of ubiquitin C-terminal hydrolase ligase-1 (UCHL-1) and the levels of endothelial nitric oxide synthase (eNOS), and decreased the levels of interleukin-6 (IL-6) and inducible NOS (iNOS) expression. In summary, our study offers evidence for the anti-inflammatory potential of FLV and σ1R in experimental colitis, and our results present a promising approach to the development of new σ1R-targeted treatment options against IBD.

## 1. Introduction

Inflammatory bowel disease (IBD) is a chronic inflammatory disorder of the gastrointestinal (GI) tract, which may occur as Crohn’s disease (CD) or ulcerative colitis (UC) [1]. The main difference is the manifestation of the inflammatory damage, which in the case of UC appears only in the colon with superficial inflammation. On the contrary, CD may affect the entire GI tract from mouth to anus, with a much more serious transmural inflammation. Both disorders are characterized by remission–relapse cycles [2]. Even though it is currently under extensive research, the pathogenesis of IBD is still not precisely known.

At present, conventional treatment options against IBD are classified into 1. immunosuppressive or anti-inflammatory drugs and 2. biological therapies. These medications aim to relieve symptoms and suppress inflammation while keeping the patient in remission [3]. Current options are quite successful in treating mild cases, but are commonly ineffective against severe IBD [4]. Furthermore, long-term usage of these drugs exerts several side effects, and surgical intervention is generally inevitable in serious cases [5]. Therefore, new therapeutic targets and treatment options are urgently needed.

Several animal models are available to study IBD. From these, chemically induced colitis models are frequently applied, such as acetic acid, dextran sodium sulfate (DSS) and 2,4,6-trinitrobenzenesulfonic acid (TNBS) models [6]. TNBS-induced acute colitis was first described by Morris et al. [7]. TNBS is dissolved in ethanol and, via a single intracolonic administration to rodents, it efficiently mimics human CD with Th1 cell-driven, transmural inflammation. Ethanol not only serves as a solvent for TNBS but also disrupts the colonic barrier, allowing for TNBS to enter lower layers of the colonic wall. TNBS, as a haptenating agent, designates native colonic proteins as foreign for the immune system. This model produces an acute inflammatory state, developing ulcers within the first 24 h after induction [8]. The mentioned features enable TNBS as a potent and cost-effective tool for developing new therapeutic options against IBD.

IBD is characterized by elevated production of proinflammatory cytokines, such as interleukin-6 (IL-6) [9] and tumor necrosis factor α (TNF-α) [10]. Specific antibody therapies against these targets seem to be effective in most cases but very expensive [11]. Furthermore, nuclear mediators, such as NF-κB and high mobility group binding 1 (HMGB1), are also important in IBD as proinflammatory stimuli [12,13]. HMGB1 is suggested as a potential biomarker in IBD patients’ feces [14], while anti-HMGB-1 therapy seems to be promising in a mouse model of colitis [13]. NF-κB is a well-known regulator of inflammatory processes and has a central role in the development of Peyers’ placks, the immune sensors of the gut [15]. Moreover, several enzymes were revealed to contribute to the pathogenesis of the disease, thus serving as novel therapeutic targets. One of these is the nitric oxide synthase (NOS), especially the inducible (iNOS) and endothelial (eNOS)) isoforms [16]. The end product of these enzymes is NO, which has a dual role in the GI tract depending on its’ quantity and origin. iNOS produces excessive amounts of NO, and contributes to IBD as proinflammatory stimuli, while eNOS generate a lower amount of constitutive NO, which plays physiological roles in the gut [17]. Previous results from our laboratory suggest heme oxygenase (HO), an antioxidant enzyme, as a therapeutic target against experimental colitis as well [18,19]. Recently, it was suggested that a deubiquitinase (dub) enzyme, ubiquitin C-terminal hydrolase ligase-1 (UCHL-1, also known as protein gene product (PGP9.5)), seems to play a suppressor role in inflammation [20], besides its essential function in proteasomal degradation [21].

Sigma receptor (σR) was originally discovered by Martin et al. in 1976 as an opioid receptor [22]. Then ligand binding studies have clarified that σR possesses a unique receptor class [23]. σR is a transmembrane protein located in the mitochondria-associated ER membrane (MAM) with an essential role in the maintenance of proper Ca^2+^ homeostasis. Upon harmful stimuli, e.g., ER stress, σR can be rapidly translocated to the plasma membrane and the nuclear membrane [24]. In the ER membrane, σR forms a complex with a binding immunoglobulin protein (BiP) chaperone [25]. Currently, two subtypes of σRs have been cloned, namely σ1R and σ2R. The separation was based on their different molecular weight and ligand binding affinities [23,26]. Besides the fundamental role of σ1R in the brain, several studies have suggested its function in peripheral tissues as well, including the heart [27], kidney [28] and the GI tract [29].

σ1R is currently considered to be a therapeutic target for the treatment of neurodegenerative diseases and depression [30]. Furthermore, it is increasingly clear that σ1R may have a role against oxidative stress, ER stress [31] and inflammation [32]. Recent studies have revealed that enhanced or overexpressed σ1R has an anti-inflammatory effect in lipopolysaccharide (LPS)-induced inflammation and in a sepsis model [33]. Moreover, treatment with σ1R agonists, such as (+)-pentazocine, have been shown to suppress inflammatory responses in LPS-induced retinal microglia activation [34].

A vast number of different ligands shows high affinity for σ1R, such as bensomorphans, antipsychomimetics, antihistamins, antidepressants and atifungal agents [35]. Accordingly, testing the effects of σ1R pharmacologically is challenging, because the high diversity of ligands may exert highly diverse side effects. Fluvoxamine (FLV) is an antidepressant drug, clinically used for the treatment of anxiety disorders and depression [36]. Furthermore, it has SSRI properties [37] and has been proven to be a specific agonist for σ1R. Other selective serotonin reuptake inhibitors (SSRIs) bind to σ1R as well, in the following affinity from highest to lowest: fluvoxamine > sertraline > fluoxetine > escitalopram > citalopram >> paroxetine [38]. It has also been shown that FLV can induce activating transcription factor-4 (ATF-4) mediated expression of σ1R and exerts the alleviation of ER stress, thus contributing to neuroprotection [39]. BD1063 has been used as a potent and selective σ1R antagonist in several experimental designs, [40,41] and found to exert a proinflammatory effect in a combined treatment with a σ1R agonist in LPS-induced inflammatory condition [34].

In the current study, we hypothesized that σ1R, through the administration of FLV, would activate anti-inflammatory mechanisms in experimental IBD. According to our results, σ1R seems to induce anti-inflammatory actions after FLV treatment; therefore, we suggest the receptor as a novel target in the treatment of IBD. Furthermore, we suggest the contribution of the UCHL-1-IL-6 pathway in the protective mechanism exerted by σ1R through FLV.

## 2. Results

### 2.1. In Vitro Radioligand Binding Assays of Sigma-1 Receptor in TNBS-Induced Colitis Rat Colon Tissue

Limited information is available on σ1R binding sites in the colon and the effects of σ1R agonists in inflammatory diseases. To evaluate if the σ1R agonist fluvoxamine (FLV) exerts its anti-inflammatory action through binding to σ1R, we characterized σ1R binding sites in control and treated membranes from rat colons. The binding affinity (K_i_, K_d_) and maximum binding capacity (receptor density, B_max_) of the specific σ1R ligand (+)-pentazocine were determined using competitive and saturation binding assays, as described in the materials and methods section.

**Figure 1:**
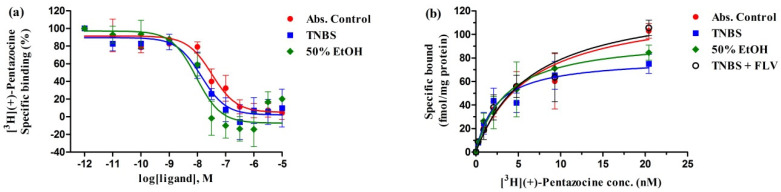
(**a**) Homologous displacement curves for (+)-pentazocine against [^3^H](+)-pentazocine (2.3 nM) binding to sigma-1 receptor (σ1R) site in control (Abs. Control) and 2,4,6-trinitrobenzenesulfonic acid-induced colitis (TNBS) or 50% ethanol enema (50% EtOH) treated rat colon membranes. (**b**) Homologous saturation isotherm for [^3^H](+)-pentazocine (0.26–20.4 nM) binding to control (Abs. Control) and TNBS-induced colitis rat colon membrane in the absence (TNBS) or presence of 1 mg/kg FLV administration (TNBS + FLV) (described in Section 4.3), 50% ethanol enema (50% EtOH) treated rat colon membranes. Curves are shown as percent specific binding ± S.E.M of at least three independent experiments. Non-specific binding was determined in the presence of 10 μM haloperidol.

The specific binding of [^3^H](+)-pentazocine was found to be saturable and of high affinity (dissociation equilibrium constants (K_d_) in the nanomolar range) in both tissue homogenates. In control (untreated) rat colon samples, moderate receptor density was observed (B_max_ = 126 ± 17). However, σ1R density was significantly decreased in TNBS-induced colitis rat colon tissue (B_max_ = 79 ± 6.5) and in the ethanol-treated group (50% EtOH). It was found that FLV treatment (1 mg/kg) significantly enhanced the maximum binding capacity (B_max_ = 134 ± 14) of the σ1Rs in TNBS-induced colitis rat colon tissue; thus, the density of σ1Rs was higher than that observed in control samples. The affinity of the radioligand did not change significantly in the treated samples (Figure 1b, Table 1).

The σ1R specific (+)-pentazocine could displace the radioligand [^3^H](+)-pentazocine from the binding site with a high binding affinity in the nanomolar range. A significant difference was observed in the inhibitory constant values of (+)-pentazocine measured in the control and treated samples. (+)-Pentazocine had a higher affinity to σ1R in TNBS-induced colitis rat colon tissue (K_i_ = 7.1 ± 1.5) and in ethanol-treated samples (K_i_ = 5.5 ± 0.9) as compared to the control rat colon homogenate (K_i_ = 24 ± 5.1) (Figure 1a, Table 1).

### 2.2. Effects of Sigma-1 Receptor on the Severity of Inflammation in TNBS Colitis

**Figure 2:**
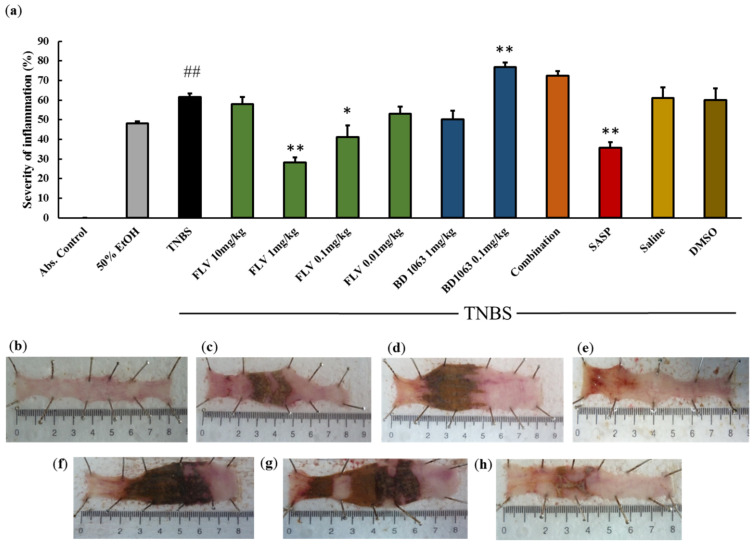
(**a**) Effects of sigma-1 receptor (σ1R) on the severity of inflammation in TNBS-induced rat colitis. The σ1R agonist, Fluvoxamine (FLV) (1 mg/kg dose) significantly decreased the severity of inflammation, the effect of which was abolished by the administration of a 0.1 mg/kg dose of the antagonist BD1063 (Combination). Vehicles for the different treatments: 50% ethanol (EtOH), physiological saline (Saline), 3% dimethyl sulfoxide (DMSO). Representative pictures of the effects of the different treatments: (**b**) absolute control (no treatment), (**c**) 50% EtOH enema, (**d**) 2,4,6-trinitrobenzenesulfonic acid (TNBS) enema, (**e**) TNBS + FLV 1 mg/kg dose, (**f**) TNBS + BD1063 0.1 mg/kg, (**g**) TNBS + combination of the effective doses (FLV 1 mg/kg + BD1063 0.1 mg/kg), (**h**) TNBS + SASP per os as a positive control. Data are represented in mean ± SEM, *n* = 4–14/group, * *p* < 0.05; ** *p* < 0.01 TNBS vs. TNBS + Treatment; ## *p* < 0.01 Abs. control vs TNBS.

To establish whether σ1R has an anti-inflammatory role, colitis was induced in rats by the intracolonic (i.c.) administration of 2,4,6-trinitrobenzenesulfonic acid (TNBS). As a vehicle for TNBS, ethanol caused superficial ulceration in the colonic tissue. TNBS caused more severe inflammation than ethanol, with serious ulcers, by haptenating native colonic proteins and thus targeting them as foreign. After TNBS administration, animals were treated i.c. with σ1R agonist Fluvoxamine (FLV) once a day with different doses (10 mg/kg, 1 mg/kg, 0.1 mg/kg, 0.01 mg/kg). Based on our results, 1 mg/kg and 0.1 mg/kg doses of FLV significantly attenuated the severity of inflammation compared to TNBS (28.29 ± 2.53 and 41.23 ± 5.76 vs. 61.68 ± 1.7%), but the protective effect of 1 mg/kg dose was more pronounced. With a positive control, sulfasalazine (SASP), rats were treated orally. SASP significantly decreased the severity of inflammation compared to the TNBS group (35.78 ± 2.83 vs. 61.68 ± 1.7%), the result of which was similar to the effective dose of FLV. BD1063, a σ1R antagonist, was administered i.c. (1 mg/kg, 0.1 mg/kg) to check the effects of a reduced σ1R activity. As was observed 0.1 mg/kg dose of BD1063 significantly exacerbated inflammation compared to TNBS (76.77 ± 2.52 vs. 61.68 ± 1.7%). Therefore, we selected FLV 1 mg/kg and BD1063 0.1 mg/kg doses as effective and treated the animals with the two substances simultaneously. Co-treatment showed that the presence of BD1063 antagonist abolished the protective effect of FLV. The impacts of the vehicles alone were also tested on the severity of inflammation (FLV: 3% DMSO; BD1063: Physiological saline) and showed no statistical differences compared to the TNBS group (Figure 2a). Representative pictures of the colons are presented in Figure 2b–h.

### 2.3. Fluvoxamine Increased the Expression of Sigma-1 Receptor in TNBS-Induced Colitis

**Figure 3:**
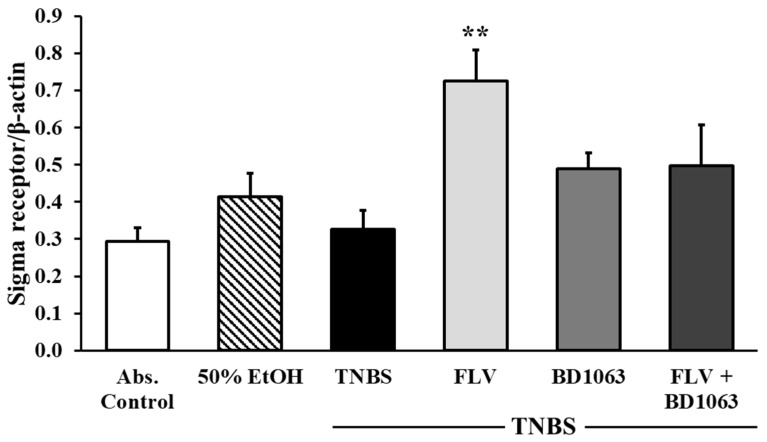
Alteration of the expression of sigma-1 receptor (σ1R) by the administration of the agonist and antagonist. Abs. control (no treatment), 50% EtOH (50% ethanol enema), TNBS (2,4,6-trinitrobenzenesulfonic acid enema), FLV (TNBS enema + 1 mg/kg fluvoxamine (FLV)), BD1063 (TNBS enema + 0.1 mg/kg BD1063), FLV + BD1063 (TNBS enema + 1 mg/kg FLV + 0.1 mg/kg BD1063). Data are represented as mean ± SEM; (*n* = 6–9); statistical significance ** *p* < 0.01 TNBS vs. TNBS + treatment.

As shown in Figure 3, the effective dose of FLV (1 mg/kg) significantly increased the expression of σ1R compared to the TNBS group (0.72 ± 0.083 vs. 0.326 ± 0.05 relative expression). The applied antagonist, BD1063 didn’t affect the expression of the receptor itself compared to the TNBS group. To further clarify our results, the effective doses of the agonist and antagonist were administered in combined treatment, and results showed that the antagonist abolished the effect of FLV on the expression of σ1R.

### 2.4. Sigma-1 Receptor Agonist Decreased the Activity of the Inflammatory Myeloperoxidase Enzyme

**Figure 4:**
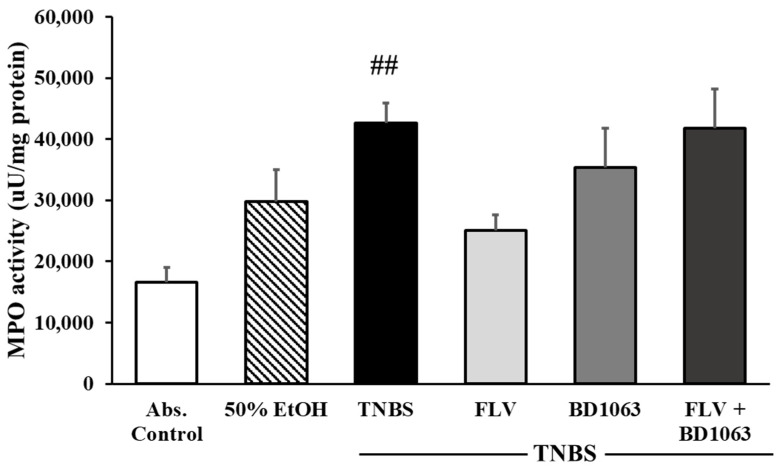
Effects of sigma-1 receptor (σ1R) on the activity of myeloperoxidase enzyme (MPO). Abs. control (no treatment), 50% EtOH (50% ethanol enema), TNBS (2,4,6-trinitrobenzenesulfonic acid enema), FLV (TNBS enema + 1 mg/kg Fluvoxamine (FLV)), BD1063 (TNBS enema + 0.1 mg/kg BD1063), FLV + BD1063 (TNBS enema + 1 mg/kg FLV + 0.1 mg/kg BD1063). Data are represented as mean ± SEM; (*n* = 4–6); statistical significance ## *p* < 0.01 Abs. control vs. TNBS.

We investigated the effects of σ1R on the activity of the inflammatory myeloperoxidase enzyme (MPO). MPO serves as an inflammatory marker, which is expressed in neutrophil granulocytes and is increased in inflammatory conditions. In our experiments, compared to control group, MPO activity was significantly increased after intracolonic administration of TNBS (16557.5 ± 2425.58 vs. 42653.4 ± 3220.24 uU/mg protein). Treatment with FLV 1 mg/kg markedly decreased the activity of the MPO enzyme compared to the TNBS group (25021.2 ± 2554.66 vs. 42653.4 ± 3220.24 uU/mg protein). Intracolonic administration of the BD1063 antagonist didn’t affect the activity of the MPO, and a combination of the two effective doses caused the same issue and abolished the effect of the agonist (Figure 4).

### 2.5. Sigma-1 Receptor Agonist Fluvoxamine Increased the Expression of UCHL-1 in the Colon

**Figure 5:**
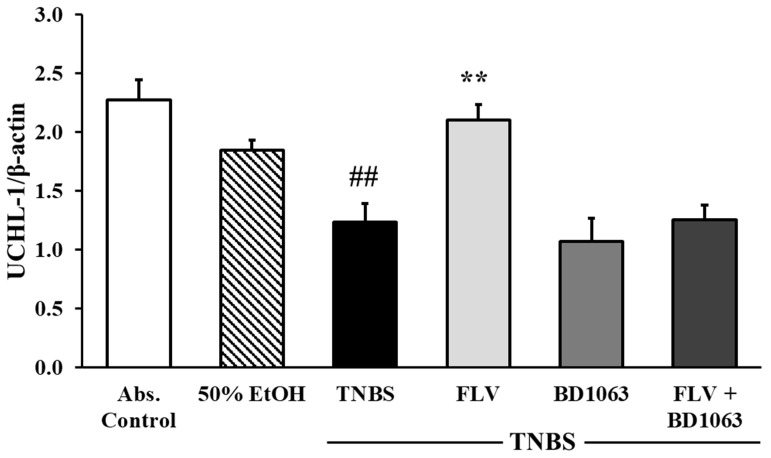
Changes in ubiquitin C-terminal hydrolase ligase-1 (UCHL-1) expression due to the administration of sigma-1 receptor (σ1R) agonist and antagonist. Abs. control (no treatment), 50% EtOH (50% ethanol enema), TNBS (2,4,6-trinitrobenzenesulfonic acid enema), FLV (TNBS enema + 1 mg/kg fluvoxamine (FLV)), BD1063 (TNBS enema + 0.1 mg/kg BD1063), FLV + BD1063 (TNBS enema + 1 mg/kg FLV + 0.1 mg/kg BD1063). Data are represented as mean ± SEM; (*n* = 5–6); statistical significance ## *p* < 0.01 abs. control vs. TNBS; ** *p* < 0.01 TNBS vs. TNBS + treatment.

To test the effects of σ1R on the expression of UCHL-1, Western blot analysis was performed. Our results showed that TNBS administration significantly decreased the expression of UCHL-1 compared to the absolute control group (2.27 ± 0.17 vs. 1.23 ± 0.16 relative expression). Treatment with FLV (1 mg/kg) significantly increased the expression of UCHL-1 compared to TNBS (2.104 ± 0.13 vs. 1.23 ± 0.16 relative expression), the effect of which was abolished by the administration of BD1063 antagonist alone or in combination with the agonist. We concluded, based on the combined treatment, that σ1R antagonist BD1063 eliminated the protective effect of FLV (Figure 5).

### 2.6. Sigma-1 Receptor Agonist Attenuated the Levels of IL-6 in the Colon

**Figure 6:**
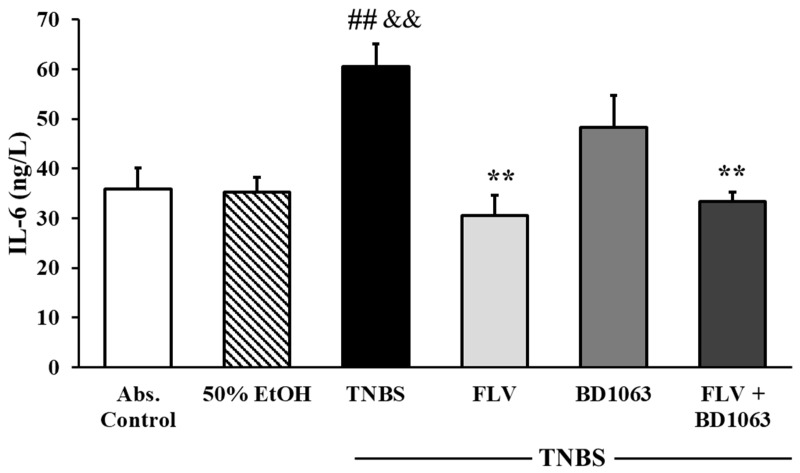
Effects of sigma-1 receptor (σ1R) agonist and antagonist on the levels of interleukin-6 (IL-6). Abs. control (no treatment), 50% EtOH (50% ethanol enema), TNBS (2,4,6-trinitrobenzenesulfonic acid enema), FLV (TNBS enema + 1 mg/kg Fluvoxamine (FLV)), BD1063 (TNBS enema + 0.1 mg/kg BD1063), FLV + BD1063 (TNBS enema + 1 mg/kg FLV + 0.1 mg/kg BD1063). Data are represented as mean ± SEM; (*n* = 6–8); statistical significance ## *p* < 0.01 Abs. control vs. TNBS; ** *p* < 0.01 TNBS vs. TNBS + Treatment; && *p* < 0.01 EtOH vs. TNBS.

IL-6 is one of the crucial proinflammatory cytokines demonstrated to be related to IBD pathogenesis. Measurement of IL-6 levels was performed by a specific double-sandwich ELISA method. The induction of colitis by TNBS administration significantly attenuated IL-6 levels compared to the absolute control group, and likewise to the 50% EtOH vehicle group (35.84 ± 4.29 vs. 60.48 ± 4.55; 35.26 ± 2.96 vs. 60.48 ± 4.55 ng/L). To evaluate the effect of σ1R on the levels of IL-6, we treated the animals with the agonist, antagonist alone and in combination as well. Treatment with the effective dose of FLV (1 mg/kg) significantly dampened the levels of IL-6 compared to the TNBS group (30.53 ± 4.10 vs. 60.48 ± 4.55 ng/L). σ1R antagonist BD1063 didn’t affect IL-6 levels compared to the TNBS group; however, combined treatment showed a significant attenuation on IL-6 levels compared to TNBS (33.45 ± 1.82 vs. 60.48 ± 4.55 ng/L) (Figure 6).

### 2.7. Sigma-1 Receptor Agonist Elevated the Activity of the Anti-Inflammatory Heme Oxygenase Enzyme

**Figure 7:**
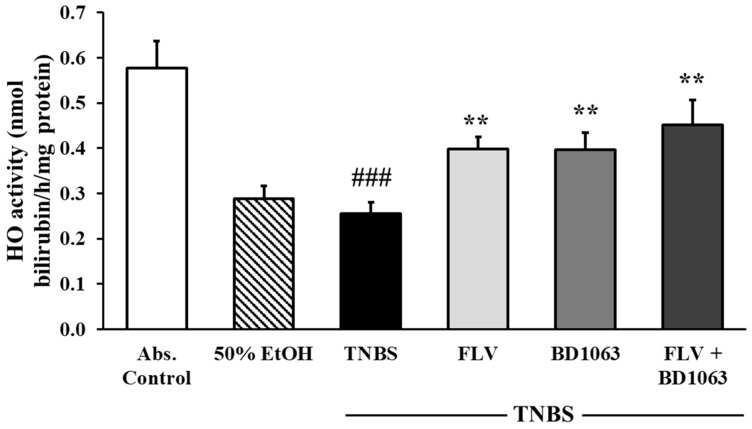
Alterations of the activity of the heme oxygenase enzyme (HO) by the presence of sigma-1 receptor (σ1R) agonist and antagonist. Abs. control (no treatment), 50% EtOH (50% ethanol enema), TNBS (2,4,6-trinitrobenzenesulfonic acid enema), FLV (TNBS enema + 1 mg/kg fluvoxamine (FLV)), BD1063 (TNBS enema + 0.1 mg/kg BD1063), FLV + BD1063 (TNBS enema + 1 mg/kg FLV + 0.1 mg/kg BD1063). Data are represented as mean ± SEM; (*n* = 5–7); statistical significance ### *p* < 0.001 Abs. control vs. TNBS; ** *p* < 0.01 TNBS vs. TNBS + treatment.

Heme oxygenase (HO) is an anti-inflammatory enzyme and has a protective role in IBD pathogenesis through the alleviation of inflammation. As shown in Figure 7, we investigated the activity of the HO enzyme by measuring the forming bilirubin content in the samples under proper conditions for the enzyme. By evaluating the results, we found significantly reduced enzyme activity in the TNBS group compared to the absolute control (0.58 ± 0.06 vs. 0.26 ± 0.03 nmol bilirubin/h/mg protein). Treatment with the effective dose of FLV (1 mg/kg) significantly increased the activity of the HO enzyme compared to TNBS (0.40 ± 0.03 vs. 0.26 ± 0.03 nmol bilirubin/h/mg protein). Surprisingly, we observed the same alteration in HO activity after the intracolonic administration of BD1063 antagonist (0.1 mg/kg) and upon combined treatment with FLV and BD1063 as well, which were also significant compared to TNBS group (0.40 ± 0.04 and 0.45 ± 0.06 vs. 0.26 ± 0.03 nmol bilirubin/h/mg protein).

### 2.8. Sigma-1 Receptor Agonist Increased the Levels of eNOS and Decreased the Expression of iNOS in the Colon

**Figure 8:**
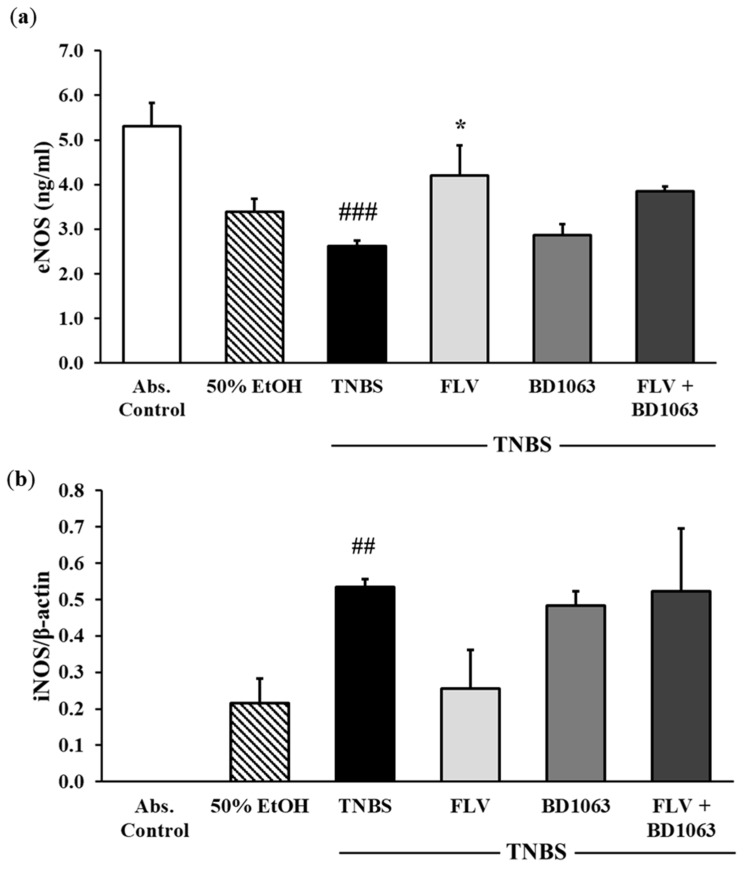
Effects of sigma-1 receptor (σ1R) agonist and antagonist on the levels of endothelial nitric oxide synthase (eNOS) (**a**) and expression of the inducible (iNOS) form (**b**). Abs. control (no treatment), 50% EtOH (50% ethanol enema), TNBS (2,4,6-trinitrobenzenesulfonic acid enema), FLV (TNBS enema + 1 mg/kg fluvoxamine (FLV)), BD1063 (TNBS enema + 0.1 mg/kg BD1063), FLV + BD1063 (TNBS enema + 1 mg/kg FLV + 0.1 mg/kg BD1063). Data are represented as mean ± SEM; (*n* = 5–8); statistical significance ## *p* < 0.01 Abs. control vs. TNBS; ### *p* < 0.001 Abs. control vs. TNBS; * *p* < 0.05 TNBS vs. TNBS + treatment.

We determined two isoforms of the NOS enzyme. Endothelial NOS (eNOS) showed a significant attenuation after TNBS treatment compared to control group (5.31 ± 0.52 vs. 2.62 ± 0.126 relative expression). Treatment with the effective dose of FLV (1 mg/kg) significantly increased the levels of eNOS (4.21 ± 0.66 vs. 2.62 ± 0.126 relative expression), the effect of which was abolished by the administration of BD1063 antagonist, and after combined treatment as well (Figure 8a). Through the measurement of the proinflammatory inducible NOS (iNOS) isoform by Western blotting, we found elevated expression of this enzyme, which was significant compared to absolute control (0 vs. 0.53 ± 0.021 relative expression). FLV markedly reduced the expression of iNOS compared to TNBS alone (0.255 ± 0.11 vs. 0.53 ± 0.02 relative expression). BD1063 antagonist did not alter the expression of iNOS compared to TNBS. Furthermore, in the case of FLV and BD1063 combined administration, iNOS remained on similar expression levels to TNBS alone (Figure 8b).

### 2.9. Sigma-1 Receptor Agonist Decreased the Expression of NF-κB p65 Subunit

**Figure 9:**
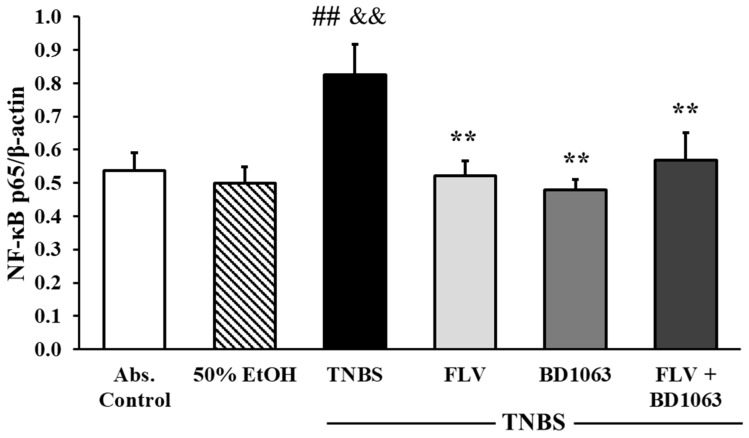
Effects of sigma-1 receptor (σ1R) agonist and antagonist on the expression of NF-κB p65 subunit. Abs. control (no treatment), 50% EtOH (50% ethanol enema), TNBS (2,4,6-trinitrobenzenesulfonic acid enema), FLV (TNBS enema + 1 mg/kg fluvoxamine (FLV)), BD1063 (TNBS enema + 0.1 mg/kg BD1063), FLV + BD1063 (TNBS enema + 1 mg/kg FLV + 0.1 mg/kg BD1063). Data are represented as mean ± SEM; (*n* = 5–9); statistical significance ## *p* < 0.01 Abs. control vs. TNBS; ** *p* < 0.01 TNBS vs. TNBS + treatment; && *p* < 0.01 EtOH vs. TNBS.

As shown in Figure 9, the induction of colitis by TNBS significantly elevated the expression of the NF-κB p65 subunit compared to the absolute control and the 50% EtOH vehicle group as well (0.54 ± 0.05 and 0.5 ± 0.05 vs. 0.83 ± 0.09 relative expression). Treatment with FLV significantly attenuated the expression of p65 compared to the TNBS group (0.52 ± 0.04 vs. 0.83 ± 0.09 relative expression). Interestingly, BD1063 was also shown to significantly reduce the expression of p65; the same phenomenon was observed in animals which were treated with the combination of the effective doses, and the result was also significant compared to TNBS (0.48 ± 0.03 and 0.57 ± 0.08 vs. 0.83 ± 0.09 relative expression).

### 2.10. Sigma-1 Receptor Agonist Decreased the Expression of HMGB1 in the Colon

**Figure 10:**
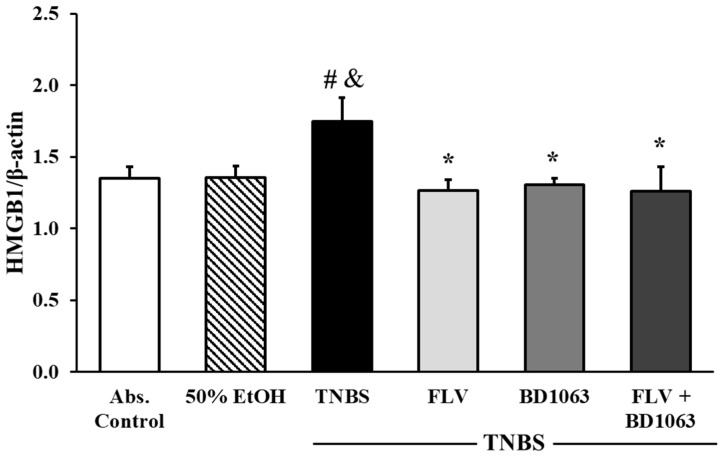
Changes in the expression of high mobility group box 1 (HMGB1), through the administration of sigma-1 receptor (σ1R) agonist and antagonist. Abs. control (no treatment), 50% EtOH (50% ethanol enema), TNBS (2,4,6-trinitrobenzenesulfonic acid enema), FLV (TNBS enema + 1 mg/kg fluvoxamine (FLV)), BD1063 (TNBS enema + 0.1 mg/kg BD1063), FLV + BD1063 (TNBS enema + 1 mg/kg FLV + 0.1 mg/kg BD1063). Data are represented as mean ± SEM; (*n* = 5–6); statistical significance # *p* < 0.05 Abs. control vs. TNBS; * *p* < 0.01 TNBS vs. TNBS + treatment; & *p* < 0.05 EtOH vs. TNBS.

The expression of HMGB1 was measured by Western blotting. Our results show that HMGB1 was significantly overexpressed in TNBS-induced colitic animals’ colons compared to the absolute control (1.35 ± 0.08 vs. 1.75 ± 0.165 relative expressions) and the ethanol group as well (1.36 ± 0.07 vs. 1.75 ± 0.165 relative expressions). The effective dose of the agonist (FLV, 1 mg/kg) significantly reduced the expression of this parameter (1.264 ± 0.07 vs. 1.75 ± 0.165 relative expressions) and, interestingly, we found the same significant attenuation in the case of BD1063 antagonist alone (1.305 ± 0.044 vs. 1.75 ± 0.165 relative expressions) and the combination of the two effective doses as well, compared to TNBS (1.258 ± 0.17 vs. 1.75 ± 0.165 relative expressions) (Figure 10).

## 3. Discussion

IBD is considered an autoimmune disorder, which manifests in predisposed hosts through an overreaction to the normal microbiome or one’s own GI cells and molecules [42]. Therapeutic options for IBD are symptomatic, usually aiming to halt proinflammatory and inflict anti-inflammatory processes [43]. In our study, animals received intracolonic TNBS as an induction method of experimental colitis. Then, rats were treated with selective σ1R agonist, fluvoxamine (FLV) and antagonist (BD1063). The results show evidence for the anti-inflammatory effect of FLV as a σ1R agonist; serving the receptor may be a novel candidate for the treatment of IBD.

Research on σ1R is mainly focused on the CNS, but it is increasingly clear that the receptor has a wider role and it is essentially important in peripheral tissues as well. Our radioligand competition and saturation binding studies revealed the existence of [^3^H](+)-pentazocine labeled σ1R binding sites with moderate density in rat colon tissue. According to Hara et al. [44], σ1R is mainly localized in the mucosa and submucosal plexus, and ligands have been found to promote alkaline secretion in the GI tract. Several types of ligands show a high affinity for σ1R [35]. Based on this high diversity, σ1R agonists and antagonists are hard to distinguish and their activities are still controversial. It is suggested that ligands of σ1R exert their effects in two possible ways: regulation of the BiP-σ1R complex and control of the oligomerization state of the receptor. Upon binding to σ1R, agonists facilitate the dissociation of BiP, an ER located chaperone, thus contributing to receptor activation, and antagonists tend to stabilize the σ1R-BiP complex [45]. Furthermore, agonists seem to promote the formation of monomers and dimers, the active forms of the receptor, as opposed to antagonists, which facilitate the formation of higher state oligomers and keep the receptor in an inactive form [46]. We found that FLV (1 mg/kg) treatment significantly enhanced the maximum binding capacity (B_max_ = 134 ± 14) of σ1Rs in rat colon tissue in TNBS-induced colitis; thus, the density of σ1Rs was higher than what was observed in control samples. Furthermore, we found a significantly higher expression of σ1Rs in response to the agonist, while the presence of BD1063 antagonist abolished the effect of FLV. Our results are in accordance with Omi et al. [39], who found the same alteration in the expression of σ1R due to FLV treatment in vitro. Interestingly, Zhao et. al. [34], using another σ1R agonist, (+)-pentazocine, didn’t find any change in the expression level of σ1R in LPS-induced inflammatory condition. Based on our aforementioned results, we presume that the role of σ1R in the colon is remarkable, and that FLV seems to exert its effects via σ1R.

**Figure 11:**
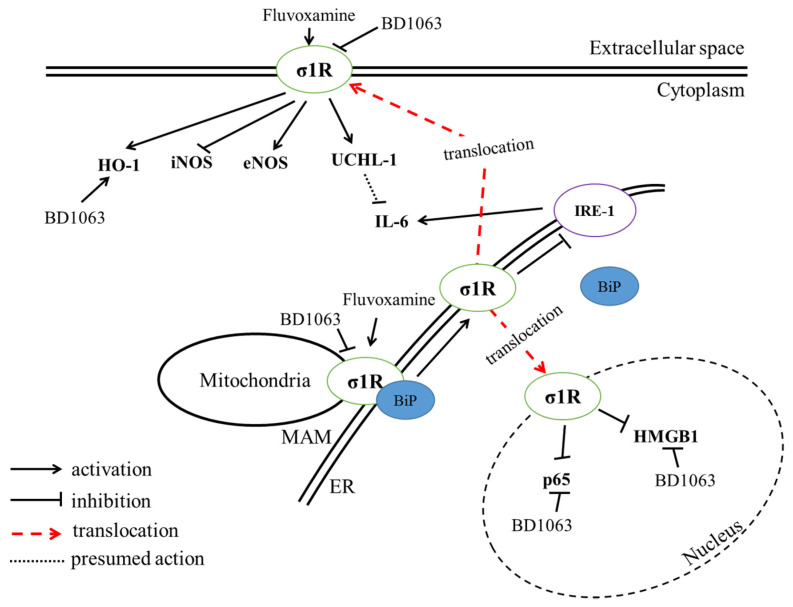
Summary of the suggested action of Sigma-1 receptor (σ1R) agonist, fluvoxamine, and antagonist, BD1063. Heme oxygenase-1 (HO-1); inducible nitric oxide synthase (iNOS); endothelial NOS (eNOS); ubiquitin C-terminal hydrolase ligase (UCHL-1); interleukin-6 (IL-6); endoplasmic reticulum (ER); mitochondria-associated ER-membrane (MAM); binding immunoglobulin protein (BiP); inositol-requiring enzyme 1α (IRE-1), p65 (NF-κB subunit); high mobility group box-1 (HMGB-1).

One of the main symptoms of IBD is the formation of GI ulcers [2]. Our data demonstrate that the effective dose of FLV significantly decreased the extent of the colonic inflammation. The protection was as effective as one of the clinically used treatments, SASP, which unfortunately has several side effects [47]. Numerous antidepressant medications have been proven to exert anti-ulcerative effects. Serotonin (5-HT) has essential roles in gut motility and normal function [48]. However, IBD and related animal models are characterized by elevated production of 5-HT. A high amount of 5-HT may serve as a proinflammatory signal, and this elevation seems to be pivotal for the development of experimental IBD [49]. According to Ghia et al. [50], inhibition of 5-HT synthesis locally in the gut by parachlorophenylalanine seems to protect against DSS-colitis. FLV has SSRI properties and may increase the availability of 5-HT in the extracellular space via binding to serotonin reuptake transporters (SERT). Linden et al. [51] found, however, that the administration of fluoxetine, another SSRI, didn’t affect 5-HT levels in the gut because of a reduced expression of SERT is associated with TNBS-induced inflammation. The authors suggested that, in physiological conditions, SSRIs may increase the availability of 5-HT in the gut via SERTs, but, as inflammation is associated with a reduced SERT expression, SSRIs have potentially lower efficacy in modulating 5-HT levels in such conditions. However, since the literature is quite controversial on the exact role of 5-HT in IBD, it cannot be completely excluded that altered 5-HT metabolism by FLV may contribute to the demonstrated anti-inflammatory effect. As a possible clarification and to further ascertain the involvement of σ1R in FLV-induced protection, we tested the effect of FLV in the presence of a σ1R antagonist as well. The results confirmed our hypothesis and showed that BD1063 antagonist abolished the protective effect of FLV. This phenomenon is in accordance with the findings of Elsaed et al. [52]. They showed that FLV treatment per os significantly decreased the extent of gastric ulcers in a rat model of stress-induced ulceration. Furthermore, Rosen et al. [33] suggested that the anti-inflammatory action of FLV depends on σ1R, and they presumed the role of inositol-requiring enzyme 1α (IRE-1), an ER-localized proinflammatory mediator in its action. To establish the molecular mechanism behind FLV-induced anti-inflammatory actions, we analyzed pro- and anti-inflammatory parameters. A summary of our findings is shown in Figure 11.

In addition, maintaining normal blood flow in the gut mucosa seems to be an important factor in experimental ulcer models [53]. As long as normal blood flow is maintained in such noxious conditions, no or minimal ulcers occur. For instance, it was found in DSS-induced rat colitis that ghrelin may produce its antiulcerative feature by maintaining normal blood flow [54], indicating a pivotal role for normal blood flow in ulcer healing. Furthermore, this notion was also supported by treatment with obestatin, another product of ghrelin gene, in TNBS-induced colitic ulcers [55] and in acetic acid-induced rat colon ulcers [56]. Hosszu et al. [57] found that σ1R activation via FLV protects against renal ischemia-reperfusion injury through the improvement in renal blood supply. Moreover, fluoxetine was shown to increase blood flow in a rat model of stroke, interestingly, in a serotonin-independent manner [58]. To our best knowledge, since such data on FLV is unavailable in the gastrointestinal tract, it should be taken under consideration in future experiments that FLV might increase mucosal blood flow via σ1R as well, which can potentially contribute to its protective effect in ulcer healing.

UCHL-1 is a deubiquitinating (DUB) enzyme, which is increasingly proposed to play an immunosuppressive role in inflammation. Gu et al. [20] found that proinflammatory cytokines tend to increase the expression of UCHL-1 in multipotent mesenchymal stromal cells, and UCHL-1 exerts anti-inflammatory actions. Here, we found that FLV treatment significantly upregulated the expression of UCHL-1, and the presence of the antagonist counteracted the protective effect of FLV. This supports the assertion that UCHL-1 changed according to the σ1R based treatment; thus, we suggest the interaction between the receptor and UCHL-1. Furthermore, we tested the effect of σ1R on the levels of the proinflammatory cytokine, IL-6, which is especially important in the pathogenesis of IBD [9]. We found that FLV decreased the levels of IL-6 in the colon. BD1063 administration didn’t change IL-6 levels compared to TNBS but, interestingly, the combined treatment with FLV and BD1063 showed similar results to the treatment with FLV alone. In this case, we presume that the effect was σ1R dependent, but, based on the fact that the effective doses were not equal in our combined treatment, we suggest that a lower dose of FLV might also exert IL-6 reduction, even in the presence of the antagonist. Li et al. [59] suggested the downregulatory role of UCHL-1 in IL-6 proinflammatory cytokine levels in skeletal muscle, which is in an agreement with our current findings. Moreover, Rosen et al. [33] found that σ1R knockout mice have higher expression of IL-6, and FLV decreased the expression of this cytokine in an σ1R-IRE-1 dependent manner. We suggest, according to our current findings, that the FLV-induced anti-inflammatory effect presumably relied upon σ1R and UCHL-1 along with the reduction of IL-6.

MPO enzyme, commonly used as an experimental inflammatory marker, showed no change in our study with BD1063 administration compared to TNBS, but markedly decreased after treatment with FLV. Several authors, including us, proposed the anti-inflammatory HO enzyme as a therapeutic target against IBD. Previous reports from our laboratory show that the induction of HO seems to be protective in experimental colitis [18]. In accordance with these previous findings, here we report that FLV increased the activity of HO enzyme. Mendez-David et al. [60] found another SSRI, fluoxetine, as a potent inducer of HO-1 expression through the activation of nuclear factor erythroid 2–related factor 2 (Nrf2), a known HO-1 promoting transcription factor. Interestingly, we observed the same alteration with the antagonist treatment as well. Since the exact signaling mechanism is unknown in both applied σ1R ligands, we assume that BD1063 might exert an induction signal for HO enzyme as well. As a possible outcome, we refer to the work of Pal et al., who investigated the levels of reactive oxygen species (ROS) in σ1R KO mice, and found elevated ROS production compared to the wild type. Moreover, the authors found that treatment with BD1063 also increased ROS [61]. As ROS are one of the main inducers of the HO enzyme [62], we assume that the HO inducer action of BD1063 might rely on its capacity to produce ROS.

NOS enzyme is suggested as an important modulator of inflammation in IBD. iNOS produces a high amount of NO and contributes to inflammation, while eNOS continuously generates a lower amount, which is essential for proper colonic homeostasis [63]. Here we found a novel candidate for altering NOS enzymes in experimental colitis. Our results showed that FLV increased the levels of eNOS and decreased iNOS, while the presence of the antagonist abolished the protective effects of FLV in case of both isoforms. In accordance, Vagnerova et al. [64] found that (+)-pentazocine, as a σ1R agonist, was able to inhibit iNOS in the brains of C57/Bl6 mice. Furthermore, Bhuiyan et al. [28] found, in ovariectomized rats, that the administration of dehydroepiandrosterone (DHEA), a σ1R agonist, showed the ability to restore the reduced eNOS levels in hypertension-induced kidney hypertrophy.

Observing essential inflammatory mediators in the nucleus, such as NF-κB and HMGB1, our results are controversial. Interestingly, FLV was able to reduce the expression of the p65 subunit of NF-κB and HMGB1, but the presence of the antagonist showed similarly decreased expression levels in both parameters compared to FLV. According to Meunier et al. [65], knockdown of σ1R was found to induce the NF-κB pathway in a Chinese hamster ovary cell culture. Furthermore, Hyrskyluoto et al. [66] observed an elevation in p65 expression in neuronal PC6.3 cells after the administration of a PRE084 σ1R agonist. Zhang et al. [67] found that methamphetamine, a known σ1R regulated drug, elevated the expression of HMGB-1 and increased the translocation of p65 to the nucleus. They also found that pretreatment with a σ1R antagonist, BD1047, decreased the translocation of the p65 subunit. We suggest that the discrepancy between these results and ours is potentially based on the fact that in vivo application of these drugs might exert distinct effects compared to in vitro studies. Moreover, as the exact signaling pathway behind the effect of BD1063 is not known, it is conceivable that BD1063 might promote the reduction of NF-kB and HMGB-1 expressions in a yet unknown pathway; thus, further examinations are needed.

## 4. Materials and Methods

### 4.1. Drug Preparations

(+)-Pentazocine, haloperidol, and buffer components (TRIS-HCl, inhibitors) were purchased from Sigma-Aldrich Kft. (Budapest, Hungary). The radioligand [^3^H]-(+)-pentazocine (s.a. 1.98 TBq/mmol) was prepared in the Laboratory of Chemical Biology (BRC, Szeged, Hungary). Tritium labeling was carried out in a self-designed vacuum manifold [68] and radioactivity was measured with a Packard Tri-Carb 2100 TR liquid scintillation analyzer (Packard, Perkin Elmer, Waltham, MA, USA) using Insta Gel scintillation cocktail of PerkinElmer. Drugs were dissolved at 1 mM in dimethyl sulfoxide (DMSO) and stored at −20 °C, and then diluted in the binding buffer. 2,4,6-trinitrobenzenesulfonic acid (TNBS) was purchased from Sigma-Aldrich (Budapest, Hungary), and was dissolved in 50% ethanol and distilled water mixture. Fluvoxamine (Fluvoxamine maleate, Sigma-Aldrich, Budapest, Hungary) was dissolved in 3% dimethyl sulfoxide (DMSO). BD1063, purchased from Tocris (Bio-Techne R&D Systems Kft., Budapest, Hungary), was dissolved in physiological saline (0.9%). Sulfasalazine (SASP) was used as a positive control and purchased from Sigma-Aldrich (Budapest, Hungary). The anesthetic agent Thiopental (Tiobarbital Braun, 0.5 g, B. Braun Medical SA, Barcelona, Spain) was dissolved in saline (0.9%).

### 4.2. Radioligand Binding Assays of Sigma-1 Receptor

#### 4.2.1. Preparation of Rat Colon Membrane Homogenates

All manipulations were performed in accordance with the standards of the European Community guidelines for the Care and Use of Laboratory Animals, and were approved by the Institutional Ethics Committee (XX./4799/2015, 15 December 2015) at the University of Szeged. The researchers made the best effort to minimize the number of animals used and ensure their wellbeing. Male Wistar–Harlan rats (225–250 g, Toxicoop Ltd. (Dunakeszi, Hungary) were used for the preparation of rat colon homogenates, which was performed according to a previous method [69], with a slight modification. Euthanized rats (Thiopental, i.p. 100 mg/kg) were killed by decapitation, and their colons were removed rapidly. Minced fresh colons were homogenized in ice-cold homogenization buffer (10 mM NaH_2_PO_4_ pH 7.4, 0.32 M sucrose, 1 mM MgSO_4_, 10 μg/mL leupeptin, 1 μg/mL pepstatin A, 5 μg/mL soybean trypsin inhibitor, 0.5 mM EGTA, 1 mM AEBSF) using a Braun Teflon-glass homogenizer at the highest rpm for 30 s. The homogenate was centrifuged at 17,000× *g* for 10 min (4 °C). The supernatant was recentrifuged at 100,000× *g* for 60 min at 4 °C. The resulting pellet was resuspended in 10 volumes homogenization buffer, homogenized with a glass homogenizer and stored in aliquots at −80 °C. The protein content of the samples was measured by the Bradford method, and samples were diluted to obtain the appropriate amount for the assay.

#### 4.2.2. Radioligand Binding Assays

Binding assays for the sigma-1 receptor were performed at 37 °C for 120 min in a 50 mM Tris–HCl binding buffer (pH 8.0) in plastic tubes in a total assay volume of 1 mL that contained 0.6 mg/mL of a membrane protein. Competition binding experiments were carried out by incubating rat colon membranes with 2.3 nM of [^3^H](+)-pentazocine (K_d_ = 6.4 nM) in the presence of increasing concentrations (10^−11^–10^−5^ M) of various competing unlabeled ligands. Non-specific binding was determined in the presence of 10 µM of haloperidol. The equilibrium dissociation constant (K_d_) and the maximum number of binding sites (B_max_) were determined by saturation binding experiments performed with increasing concentrations of [^3^H](+)-pentazocine (0.26–20.4 nM) in the absence (total binding) or presence (non-specific binding) of 10 μM haloperidol. The incubation was terminated by diluting the samples with an ice-cold wash buffer (50 mM of Tris–HCl, pH 8.0), followed by repeated washing and rapid filtration through Whatman GF/B glass fiber filters (Whatman Ltd., Maidstone, UK) presoaked with 0.1% polyethyleneimine. Filtration was performed with a 24-well Brandel Cell Harvester (Gaithersburg, MD, USA). Filters were air-dried and immersed into Ultima Gold MV scintillation cocktail, and then radioactivity was measured with a TRI-CARB 2100TR liquid scintillation analyzer (Packard, Perkin Elmer, Waltham, MA, USA).

### 4.3. Experimental Animals for the Induction of Colitis

Male Wistar–Harlan rats (225–250 g) were purchased from Toxicoop Ltd. (Dunakeszi, Hungary) and housed in a room with acclimatized temperature under 12h day/night cycles with food and water ad libitum. Animals were randomly divided into 3 groups: absolute control (no treatment, *n* = 12), 50% EtOH (ethanol enema, *n* = 12), and TNBS (10 mg dissolved in 50% Ethanol, *n* = 85). Colitis was assessed following Morris’ method [7]. Animals fasted overnight and a TNBS enema was administered intracolonically (i.c.) with an 8cm long polyethylene canulla through the anus under mild anesthesia (Thiopental, i.p. 40 mg/kg). Then, animals with TNBS-induced colitis were divided into ten groups (*n* = 6–14/group) and further treated once a day with the following drugs: fluvoxamine (sigma-1 receptor agonist) i.c. administration at different doses (10 mg/kg, 1 mg/kg, 0.1 mg/kg, 0.01 mg/kg dissolved in 3% DMSO); BD1063 (sigma-1 receptor antagonist) 1 mg/kg, 0.1 mg/kg (dissolved in physiological saline (0.9%)), FLV+BD1063 (combined administration of the two effective doses (FLV 1 mg/kg + BD1063 0.1 mg/kg)), saline (vehicle of BD1063), DMSO (3%, vehicle of Fluvoxamine) and SASP (positive control (2 × 25 mg/kg), administered per os). Animals were fasted for 5 h each day before i.c. treatments.

After 72 h of TNBS administration, euthanized animals (Thiopental, i.p. 100 mg/kg) were sacrificed and the last 8 cm portion of the colon was removed, gently opened, rinsed in physiological saline and photographed for further macroscopic analysis. Then, the colon segments were frozen in liquid nitrogen. Frozen colon tissues were powdered in liquid nitrogen by using a porcelain mortar and pestle and kept at −80 °C until used for biochemical measurements.

### 4.4. Damage Score and Measurement of the Lesions

The extent of macroscopically apparent inflammation, ulceration and tissue disruption was determined in a randomized manner from the colored images, using proprietary computerized planimetry software which was developed in our laboratory (Stat_2_1_1, Szeged, Hungary) and is based on planimetrics. The area of macroscopically visible mucosal damage was calculated and expressed as a % of the total studied 8 cm colonic segment.

### 4.5. Measurement of the Activity of MPO Proinflammatory Enzyme

Myeloperoxidase is a proinflammatory enzyme which is commonly used as a marker for inflammation and neutrophil granulocyte accumulation. 30 mg from the powdered colonic samples was measured and homogenized using a Benchmark Scientific Handheld homogenizer D1000 (Benchmark Scientific, New Jersey, MA, USA) (2 × 10 sec) in ice-cold phosphate buffer (50 mM, pH 6.0) containing 0.5% hexadecyltrimethylammonium-bromide (HETAB). To further break the membranes, three freeze–thaw cycles were applied in liquid nitrogen and a 37 °C water bath. Homogenates then were centrifuged at 10,000× *g* for 15 min at 4 °C. After collecting the supernatant, a 12 µL aliquot was mixed with 280 µL phosphate buffer (50 mM, pH 6.0) containing 0.167 mg/mL O-adenosine dihydrochloride (Sigma-Aldrich, Budapest, Hungary) and pipetted into a 96-well plate. The reaction was started with the addition of 10 *µ*L 0.03% hydrogen peroxide (H_2_O_2_). After shaking for 90 s, MPO activity was assayed spectrophotometrically at 490 nm (Benchmark Microplate Reader, Bio-Rad Laboratories, Hercules, CA, USA). MPO activity was expressed as uU/mg protein.

### 4.6. Western Blot Analyses of the Expression of Sigma-1 Receptor, UCHL-1, iNOS, NF-κB p65 and HMGB1

In total, 30 mg from each powdered sample was measured and suspended in RIPA buffer (Merck Millipore, Burlington, MA, USA) supplemented with phenylmethylsulfonyl fluoride (PMSF) (Sigma-Aldrich, Budapest, Hungary); 1/10 of the final volume. After 3 × 10 sec of homogenization using a Ultrasonic Homogenizer UP-100H (Hielscher Ultrasonics, Teltow, Germany) on ice, homogenates were centrifuged at 14,000× *g* for 10 min at 4 °C. The supernatant was collected and protein concentration was determined by using a Bradford assay and bovine serum albumin as a standard. 50 ug of each sample was loaded onto 10% sodium dodecyl sulfate (SDS)-polyacrylamide gels (8% in the case of iNOS) and run at 90V for 2 h. Gels then were transferred to nitrocellulose membranes for 2.5 h on 35V (in case of iNOS: overnight, 4 °C, 25V). Membranes were dyed with Ponceau and, after washing in TBS-T (pH 7.4), membranes were blocked in 5% milk or 5% BSA. Blots then were washed for 3 × 10 min in TBS-T and probed with first antibodies: anti-Sigma-1 receptor (Santa-Cruz Biotechnology, Dallas, TX, USA, sc-137075, 1:250), anti-UCHL-1 (Abcam, Cambridge, UK, ab108986, 1:500), anti-iNOS (Abcam, Cambridge, UK, ab3523, 1:500), anti-NF-κB p65 (Abcam, Cambridge, UK, ab16502, 1:1000) and anti-HMGB1 (Abcam, Cambridge, UK, ab79823, 1:1000) antibodies. All membranes were incubated with the first antibody for two hours at room temperature, except iNOS (overnight at 4 °C). Secondary anti-rabbit (DAKO Agilent, Santa Clara, CA, USA) and anti-mouse antibodies (DAKO Agilent, Santa Clara, CA, USA) conjugated with horseradish peroxidase were chosen properly for anti-rabbit and anti-mouse first antibodies. Incubation with the secondary antibodies lasted for 1 h at room temperature; dilution 1:5000. Signals were developed using an enhanced chemiluminescence system (ECL Plus, Amersham Pharmacia Biotech., Buckinghamshire, UK). Results were analyzed using Quantity One Software version 4.5 (Bio-Rad Laboratories, Hercules, CA, USA). Each membrane was stripped and used for the detection of β-actin as a loading control (first ab: anti-β-actin, Abcam, Cambridge, UK, ab20272, 1:10,000; second ab: anti-mouse antibody conjugated with horseradish peroxidase, DAKO Agilent, Santa Clara, CA, USA, 1:5000). Results are shown as relative expressions, normalized to β-actin.

### 4.7. Determination of IL-6, eNOS Levels in the Colon by ELISA

To determine the tissue levels of IL-6 and eNOS in the colon, we used double-antibody sandwich ELISA kits specific for rat IL-6 or eNOS. eNOS kit was purchased from MyBioSource (MBS721860; San Diego, CA, USA) and IL-6 was purchased from GenAsia Biotech Co., Ltd. (Shanghai, China). Samples were homogenized in the same homogenization buffer (Phosphate Buffer Saline (PBS), pH 7.4) and through the same homogenization procedure (Benchmark Scientific Handheld homogenizer D1000 (Benchmark Scientific, New Jersey, MA, USA); 2 × 10 sec; centrifugation: 3000 rpm, 20 min, 4 °C). The whole sample preparation procedure was done on ice. Parameters were measured according to the manufacturer’s instructions and protocols, and optical densities (OD) were assayed at λ = 450 nm. Results are expressed in ng/L (IL-6) and ng/mL (eNOS).

### 4.8. Measuring the Activity of HO Anti-Inflammatory Enzyme

Heme oxygenase activity was assayed as described by Tenhunen et al. (1968) [70] with slight modifications [71]. The assay is based on the measurement of bilirubin formation. 30 mg of each sample was measured and homogenized (Benchmark Scientific Handheld homogenizer D1000 (Benchmark Scientific, New Jersey, MA, USA); 2 × 10 sec) in ice-cold 10 mM N-[2-hydroxyethyl] piperazine- Nʼ-[2-ethanesulfonic acid] (HEPES), 32 mM sucrose, 1 mM dithiothreitol (DTT), 0.1 mM EDTA, 10 ug/mL soybean trypsin inhibitor, 10 ug/mL leupeptin, and 2 ug/mL aprotinin, at pH 7.4. After centrifugation at 20,000× *g* for 30 min at 4 °C, supernatants were collected. Incubation was carried out in the dark at 37 °C for 60 min with a 1.5-mL final volume reaction mixture containing: 2 mM glucose 6-phosphate, 0.14 U/mL glucose 6-phosphate dehydrogenase, 15 uM heme, 150 uM b-nicotinamide adenine dinucleotide phosphate (β-NADPH), 120 ug/mL rat liver cytosol as a source of biliverdin reductase, 2 mM MgCl_2_, 100 mM potassium phosphate buffer and 150 ul of the supernatant. All reagents were purchased by Sigma-Aldrich (Budapest, Hungary). The reaction was stopped by placing the samples on ice. The level of bilirubin formed was calculated from the difference between optical densities observed at 460 and 530 nm. One unit of heme oxygenase activity was defined as the amount of bilirubin produced (nmol/h/mg protein).

### 4.9. Protein Determination

Protein concentration was measured by Bradford assay. Aliquots of 20 ul of the diluted samples (30× or 40× with distilled water) were taken and mixed with 980 µL distilled water. 200 ul of Bradford reagent was added to each sample. After mixing and 10 min of incubation, samples were measured spectrophotometrically at 595 nm and compared to bovine serum albumin standard. The protein level was expressed as mg/mL.

### 4.10. Data Representation and Statistical Analysis

All data are presented as mean ± SEM. Results acquired by Western blotting were normalized to β-actin. Statistical analysis was performed using one-way ANOVA followed by the Holm–Sidak post hoc test (SigmaPlot 12, Systat Software Inc., San Jose, CA, USA) in all measurements except the ligand binding studies. The results of the competition binding studies are reported as means ± S.E.M. of at least three independent experiments, each performed in duplicate. In competition binding studies, the inhibitory constants (K_i_) were calculated from the inflection points of the displacement curves using nonlinear least-square curve fitting and the Cheng–Prusoff equation, K_i_ = EC_50_/(1 + [ligand]/K_d_). The K_i_, K_d_, and B_max_ values were compared by one-way ANOVA, followed by Bonferroni’s multiple comparison test with GraphPad Prism 5.0 (San Diego, CA, USA). Differences were considered significant in all measurements when the *p* values were less than 0.05.

## 5. Conclusions

In conclusion, our study showed evidence for the anti-inflammatory potential of FLV and σ1R in experimental colitis. We presume that the protective effect is partially exerted through FLV-induced increased expression of UCHL-1 and eNOS, and the decreased levels of IL-6 and iNOS expression. Although σ1R binds diverse classes of pharmacological compounds (different binding affinities), it is still unclear whether other σ1R agonists can produce similar pharmacological actions via σ1R as fluvoxamine. Further investigations are needed, but our current results seem to be promising and may prompt a new indication of the clinically approved Fluvoxamine for the treatment of ulcers in IBD. Research on σ1R is generally centered on CNS and neurodegenerative diseases, but here we show evidence that σ1R might have a wider potential in peripheral tissues and inflammatory conditions as well, including IBD.

## Figures and Tables

**Table 1:** Binding properties of [^3^H](+)-pentazocine for sigma-1 receptor (σ1R) in the control and TNBS-induced colitis rat colon tissue in the absence or presence of fluvoxamine (FLV) administration. n.d.: not determined. Values are mean ± S.E.M. of minimum three experiments performed in duplicate. Statistical comparison of the displacement (K_i_) and saturation (K_d_, B_max_) binding results was performed by analysis of variance (one-way ANOVA) followed by Bonferroni’s multiple comparison test (* *p* < 0.05). An asterisk represents a significant difference between inhibitory constants (K_i_) of control (Abs. Control) vs. TNBS-induced colitis rat colon membrane (TNBS), and control (Abs. Control) vs. 50% ethanol enema (50% EtOH) treated rat colon membranes. In saturation binding assays asterisk represents a significant difference between maximum binding capacity (B_max_) of Abs. Control vs. TNBS-induced colitis rat colon membrane (TNBS); and TNBS-induced colitis rat colon membrane in the absence (TNBS) vs. presence of 1mg/kg fluvoxamine administration (TNBS + FLV).

Treatment	Competition with [^3^H](+)-Pentazocine	[^3^H](+)-Pentazocine Saturation Binding	
	K_i_ ± S.E.M. (nM)	K_d_ ± S.E.M. (nM)	B_max_ ± S.E.M. (fmol/mg)
Abs. control	24 ± 5.1	6.4 ± 2.2	126 ± 15
TNBS	7.1 ± 1.5 *	2.4 ± 0.8	79 ± 6.5*
50% EtOH	5.5 ± 0.9*	3.5 ± 0.6	96 ± 5.2
TNBS + FLV	n.d.	6.7 ± 2.1	134 ± 14*
